# Associations between social and intellectual activities with cognitive trajectories in Chinese middle-aged and older adults: a nationally representative cohort study

**DOI:** 10.1186/s13195-020-00691-6

**Published:** 2020-09-25

**Authors:** Haibin Li, Changwei Li, Anxin Wang, Yanling Qi, Wei Feng, Chengbei Hou, Lixin Tao, Xiangtong Liu, Xia Li, Wei Wang, Deqiang Zheng, Xiuhua Guo

**Affiliations:** 1 Department of Epidemiology, Beijing Chaoyang Hospital, Capital Medical University, Beijing, China; 2 Department of Epidemiology and Health Statistics, School of Public Health, Capital Medical University, No. 10 Xitoutiao, You’anmen Wai, Fengtai District, Beijing, 100069 China; 3 Beijing Municipal Key Laboratory of Clinical Epidemiology, Capital Medical University, Beijing, China; 4 Department of Epidemiology, Tulane University School of Public Health and Tropical Medicine, 1440 Canal Street, New Orleans, Louisiana 70112 USA; 5 China National Clinical Research Center for Neurological Diseases, Beijing Tiantan Hospital, Capital Medical University, Beijing, China; 6 Department of Health Care Administration, College of Health and Human Services, California State University, Long Beach, 1250 Bellflower Blvd, Long Beach, CA 90840 USA; 7 Center for Evidence-Based Medicine, Xuanwu Hospital, Capital Medical University, Beijing, China; 8 Department of Mathematics and Statistics, La Trobe University, Melbourne, VIC Australia; 9 Global Health and Genomics, School of Medical Sciences and Health, Edith Cowan University, Perth, WA 6027 Australia

**Keywords:** Cognitive trajectory, Social activity, Intellectual activity, Group-based trajectory models, Longitudinal study, Aging

## Abstract

**Background:**

Associations between the frequency of social and intellectual activities and cognitive trajectories are understudied in Chinese middle-aged and older adults. We aimed to examine this association in a nationally representative longitudinal study.

**Methods:**

The China Health and Retirement Longitudinal Study (CHARLS) is a nationally representative sample of Chinese middle-aged and older participants. The frequency of social and intellectual activities was measured at baseline. Interview-based cognitive assessments of orientation and attention, episodic memory, and visuospatial skills and the calculation of combined global scores were assessed every 2 years from 2011 to 2016. Cognitive aging trajectories over time were analyzed using group-based trajectory modeling, and the associations of the trajectory memberships with social and intellectual activities were analyzed using multinomial logistic regression. Odds ratios (OR) and 95% confidence intervals (CI) were reported.

**Results:**

Among 8204 participants aged 50–75 years at baseline, trajectory analysis identified three longitudinal patterns of cognitive function based on the global cognitive scores: “persistently low trajectory” (*n* = 1550, 18.9%), “persistently moderate trajectory” (*n* = 3194, 38.9%), and “persistently high trajectory” (*n* = 3460, 42.2%). After adjustment for sociodemographic variables, lifestyles, geriatric symptoms, and health conditions, more frequent intellectual activities (OR 0.54, 95% CI 0.38–0.77) and social activities (OR 0.79, 95% CI 0.65–0.95) were both associated with a lower likelihood of being in the “persistently low trajectory” for global cognitive function.

**Conclusions:**

These findings suggested that more frequent social and intellectual activities were associated with more favorable cognitive aging trajectories.

## Introduction

Cognitive impairment and dementia are the most common geriatric symptoms in elderly individuals aged 60 years and older [1]. As the global population is aging, the number of individuals with cognitive impairment or dementia has dramatically increased both in China and internationally [2–4]. A recent meta-analysis and systematic review reported that an estimated 15% of the older Chinese population suffered from cognitive impairment [5]. A high prevalence of cognitive impairment translates into a very large economic burden [6]. There is no effective treatment for cognitive impairment or dementia [7]. Thus, early identification of potentially modifiable risk factors for cognitive decline is crucial to delay and prevent the occurrence of cognitive impairment and/or dementia [8].

A large body of studies has examined the association between leisure time activities and cognitive function in older adults [9–14]. The results have been mixed. Some studies found no association between participation in social activities and cognitive function [13, 14], while many other studies demonstrated that frequent participation in social and/or intellectual activities was associated with reduced risk for cognitive decline and dementia [9–12]. Most longitudinal studies have used linear mixed-effects models, which models correlated repeated measures with random effects, to allow individual differences in both cognitive scores at baseline and rates of cognitive decline [15, 16]. However, this strategy does not take into account the possibility that certain groups of individuals may have different developmental trajectories. Group-based trajectory modeling has been used to identify outcome patterns for cognition function. This technique is particularly useful because it has the advantage of identifying trajectories, rather than modeling the mean, which may obscure differences between groups of individuals [17]. However, very few studies have focused on the associations of social and intellectual activities with cognitive trajectories among older people.

Therefore, the present study aimed to investigate the association of intellectual and social activities with trajectory of cognitive functions by using repeated cognition measurements in a nationally representative sample of middle-aged and older Chinese adults. We hypothesized that participation in social or intellectual activity may be associated with better cognitive trajectory over time among the community Chinese elderly independently of other factors.

## Methods

### Setting

The China Health and Retirement Longitudinal Study (CHARLS) is a nationally representative study of Chinese adults aged ≥ 45 years. The CHARLS is designed to describe the dynamics of retirement and its impact on health, health insurance, and economic well-being. The baseline survey was conducted in 2011–2012 among 17,708 participants from 150 counties of China’s 28 provinces [18], and data on socioeconomic status, lifestyles, medications, health status, and functioning assessments were collected. Details on the study design, sampling procedure, and data collection have been described in previous publications [18]. Briefly, the CHARLS participants were recruited through a four-stage, stratified, cluster random sampling method. The CHARLS participants were followed biennially to obtain updated information. The CHARLS data are available for the baseline survey in 2011–2012 (wave 1), the first follow-up survey in 2013–2014 (wave 2), and the second follow-up survey in 2015–2016 (wave 3). The Biomedical Ethics Committee of Peking University approved this study, and all participants provided written informed consent.

### Study population

**Fig. 1:**
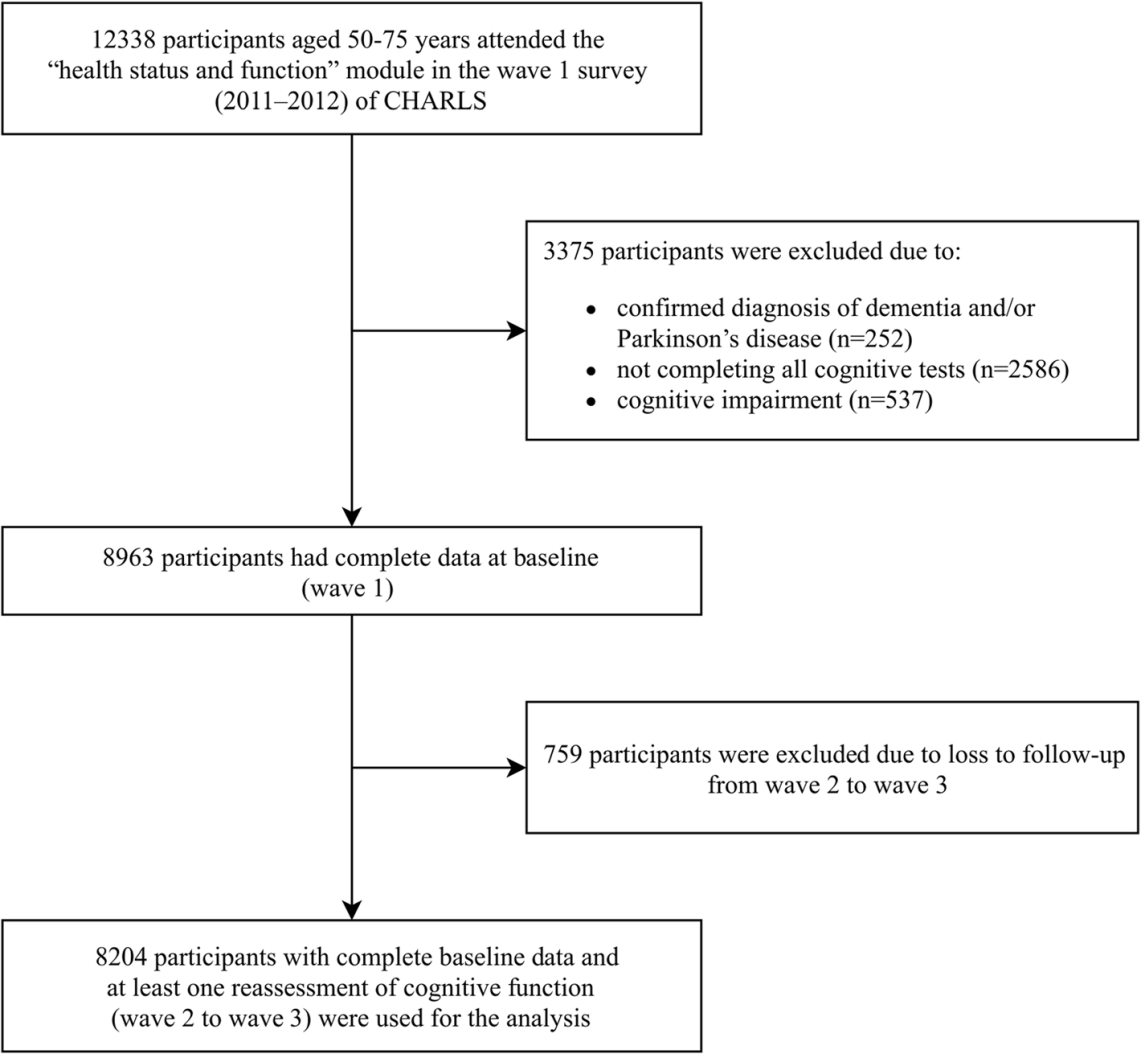
Study flow chart

The current analyses focused on 12,338 individuals who were aged 50–75 years and attended the “health status and function” module in the wave 1 survey. Of these, 3375 individuals were excluded for the following reasons: they had self-reported diagnosis of dementia and/or Parkinson’s disease (*n* = 252), they did not complete all of the cognitive tests (*n* = 2586), or they had cognitive impairment [19] (defined as a global cognitive score < 5 [1.5 SD below its mean], *n* = 537) at baseline. An additional 759 individuals were excluded because they were lost to follow-up from waves 2 to 3. The remaining 8204 participants (4289 males and 3915 females) with complete baseline data and at least one reassessment of cognitive function (waves 2–3) were included in the analyses reported here (Fig. 1).

### Social and intellectual activities

In the “health status and function” module of CHARLS, four social activities (interacting with friends; going dancing, exercising, or practicing Qigong; participating in community-related organizations; and doing voluntary charity work or assisting others) and four intellectual activities (playing Mahjong, cards, or chess; attending an educational or training course; investing in stock; and surfing the internet) in the past month were assessed. The frequency of each activity was rated as never (score = 0), not regularly (score = 1), almost every week (score = 2), or almost daily (score = 3). These activities were assembled to a sum score based on the frequency level (score 0–3). Thus, the total scores for social and intellectual activities could range from 0 to 12 points and were categorized as 0, 1–2, and ≥ 3.

### Cognitive function

In accordance with previous studies [20, 21], cognitive function was calculated using two categories: *episodic memory* and *mental intactness*. The *word recall test* evaluated *episodic memory*. Examiners read a list of 10 random words, and participants were instructed to recall as many words as possible immediately afterward (*immediate recall*). The number of correctly recalled words was scored and indicated the participant’s immediate recall. Ten minutes later, the participants were asked to recall the same list of words (*delayed recall*). *Episodic memory scores* were calculated as the average number of immediate and delayed word recalls and ranged from 0 to 10. The *mental intactness* based on some components of the mental status questions of the Telephone Interview of Cognitive Status (*TICS*) battery established to capture intactness or mental status of individuals. In CHARLS, mental status questions included serial subtraction of 7 from 100 (up to five times), the date (month, day, and year), the day of the week, the season of the year, and intersecting pentagon copying test. Answers to these questions are summed into a *mental intactness score* that ranges from 0 to 11. *Global cognitive scores* were calculated as the sum of the scores of episodic memory and mental intactness and ranged from 0 to 21.

### Covariates

Baseline measurements of age, sex, education level, marital status, location of residence, household income level, smoking, drinking, self-report of health, physician-diagnosed chronic diseases, restriction, self-reported visual and hearing impairments, depressive symptoms, and body mass index (BMI) were included as covariates in the current analyses. Educational level was categorized as “no formal education,” “primary school,” “middle school,” or “high school or above.” Marital status included “married” and “others.” Location of residence was divided into “rural” and “urban.” Household income was categorized into tertiles and coded as “low,” “medium,” and “high.” Self-perceived health status was reported as “good,” “fair,” or “poor.” Current smoking and drinking status were assessed by self-report based on the questions “Do you currently smoke?” and “Do you currently drink alcohol?” Hypertension was defined as a systolic blood pressure ≥ 140 mmHg and/or a diastolic blood pressure ≥ 90 mmHg, use anti-hypertensive drugs, or self-reported history of hypertension. Diabetes mellitus was defined as a fasting blood glucose ≥ 126 mg/dL, HbA1c ≥ 6.5%, or current use of anti-diabetic therapy, or self-reported history of diabetes mellitus. Dyslipidemia was defined as a total cholesterol ≥ 240 mg/dL, current use of lipid-lowering therapy, or self-reported history of dyslipidemia. Chronic kidney disease was defined as an estimated glomerular filtration rate < 60 mL/min/1.7 m^2^ or self-reported history of chronic kidney disease. Other physician-diagnosed chronic diseases, including heart diseases, stroke, chronic lung disease, arthritis, and cancer, were self-reported. We defined comorbidity as 0, 1, or at least 2 according to the number of nine chronic diseases that the participant had. Restriction was defined as having limitations in any of the five activities of daily living, including bathing, dressing, eating, getting into/out of bed, and toileting [22]. Depressive symptoms were assessed using the 10-item version of the Center for Epidemiologic Studies Depression Scale, and a score of ≥ 10 indicated the presence of depressive symptoms [23]. BMI was calculated as the weight in kilograms divided by the square of the height in meters and was categorized as follows: < 18.5, 18.5–23.9, 24.0–27.9, and ≥ 28.0 kg/m^2^ [24].

### Statistical analysis

The primary outcome was the trajectory of *global cognitive scores*, and the second outcomes were the trajectory of *episodic memory and mental intactness scores*. We first performed a multiple regression equation adjusting for age, sex, and education to obtain the predicted cognitive scores, and then, we used the following equation to calculate the adjusted *Z* scores: $Z=\frac{Y-{\overline{Y}}^{\prime }}{\mathrm{RMSE}}$, where *Y* is the raw cognitive score, ${\overline{Y}}^{\prime }$ is the predicted population mean score, and RMSE is the root mean square error of the regression equation [25]. We used this method to transform the global cognitive scores and scores for individual cognition domains. The transformed *Z* scores were used in analyses.

We applied group-based trajectory modeling (GBTM) implemented through the “traj” plugin procedure in Stata [26] to identify distinct trajectories of cognitive scores as a function of current age at each visit. GBTM allowed for all available cognitive scores to be included in model estimates under the assumption that missing cognitive score measures were missing at random. The successive cognitive *Z* scores were modeled as censored normal [27]. A maximum of six trajectory groups was set a priori. We fitted the models from one group trajectory to six group trajectories, and age in years was used as a timescale. To identify the model with optimal number of distinct cognitive trajectories, we first modeled longitudinal trajectories of cognitive scores by adapting a polynomial model (up to cubic models) for each of the cognitive outcomes with age as independent predictor. Then, we compared the Bayesian information criteria (BIC) and Akaike’s information criterion (AIC) value to identify the best fitted model. Furthermore, an average posterior probability of assigning each participant to a group of approximately 70% or higher was indicative of a good fit, and models with greater than 5% membership in each trajectory group were selected.

Subsequently, multinomial logistic regression model was used to estimate the association of social and intellectual activities with the trajectories of the cognitive function measures. Odds ratios (OR) and the corresponding 95% confidence intervals (CI) were reported. Multivariable-adjusted model included these following covariates: social and intellectual activity scores (0, 1–2, ≥ 3), age at baseline (continuous), sex (male, female), education (no formal education, primary school, middle or high school, college or above), marital status (married, others), residence (urban, rural), household income (low, medium, high), smoking (yes, no), drinking (yes, no), body mass index (< 18.5, 18.5–23.9, 24.0–27.9, ≥ 28.0 kg/m^2^), self-report of health (good, fair, poor), comorbidity (0, 1, ≥ 2), depressive symptoms (yes, no), restriction on activities of daily living (yes, no), visual impairment (yes, no), and hearing impairment (yes, no). The association analyses were also conducted by age group (< 65 years and ≥ 65 years) and sex (male and female) in separate models. Effect modification was tested by adding multiplicative interaction terms (i.e., social activity scores × sex) to the fully adjusted model.

All analyses were performed with Stata version 15.1 (StataCorp, College Station, TX). A two-sided *p* value less than 0.05 was considered statistically significant.

## Results

### Baseline characteristics

The mean age of the 8204 participants was 60.09 ± 6.37 years; 52.3% of participants were male. Of the sample, 22.2% of participants had a social activity score ≥ 3 and 7.4% of participants had an intellectual activity score ≥ 3. The distribution of baseline covariates and cognitive scores is shown in Table 1.

**Table 1:** Characteristic of the study cohort at baseline in middle-aged and older adults from CHARLS

Characteristic	Value (*n* = 8204)
Age (years), mean ± SD	60.09 ± 6.37
Male sex, *n* (%)	4289 (52.3)
Educational level, *n* (%)	
No formal education	3551 (43.3)
Primary school	2011 (24.5)
Middle or high school	2270 (27.7)
College or above	372 (4.5)
Married, *n* (%)	7310 (89.1)
Rural residence, *n* (%)	4906 (59.8)
High household income, *n* (%)	2351 (28.7)
Current smoker, *n* (%)	2757 (33.6)
Current drinker, *n* (%)	2856 (34.8)
Poor self-report of health, *n* (%)	2107 (25.7)
Depressive symptoms, *n* (%)	2854 (34.8)
Restriction on ADL, *n* (%)	1157 (14.1)
Visual impairment, *n* (%)	464 (5.7)
Hearing impairment, *n* (%)	600 (7.3)
Comorbidity, *n* (%)*	
0	1166 (14.2)
1	1941 (23.7)
≥ 2	5097 (62.1)
BMI (kg/m^2^), mean ± SD	23.54 ± 3.92
Social activity scores, *n* (%)	
0	4919 (60.0)
1–2	1460 (17.8)
≥ 3	1825 (22.2)
Intellectual activity scores, *n* (%)	
0	6407 (78.1)
1–2	1193 (14.5)
≥ 3	604 (7.4)
Global cognitive scores, mean ± SD	11.71 ± 3.43
Mental intactness scores, mean ± SD	7.82 ± 2.64
Episodic memory scores, mean ± SD	3.89 ± 1.61

*CHARLS* China Health and Retirement Longitudinal Study, *ADL* activities of daily living, *BMI* body mass index

*Based on nine self-reported, physician-diagnosed conditions, including hypertension, diabetes mellitus, dyslipidemia, heart diseases, stroke, chronic lung disease, chronic kidney disease, arthritis, and cancer

### Estimated cognitive aging trajectories

**Fig. 2:**
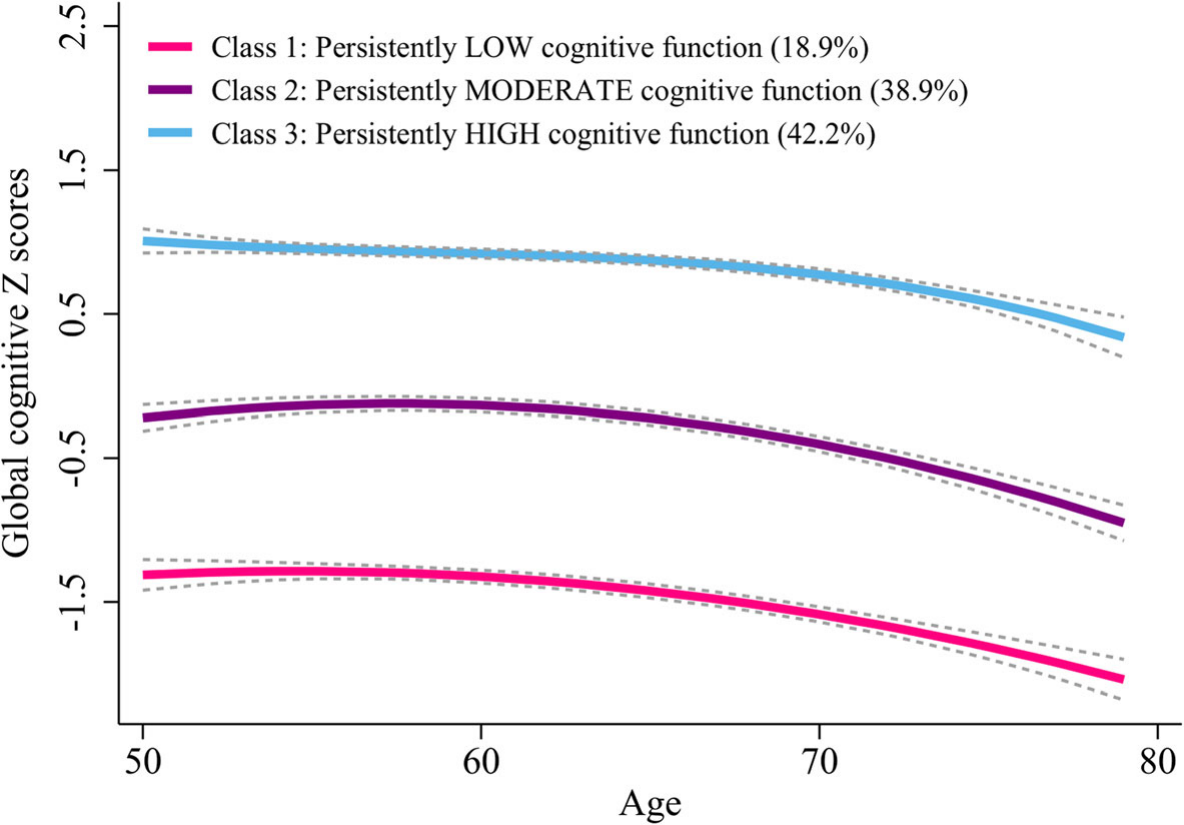
Mean trajectories of global cognitive scores by increasing age among older adults

**Fig. 3:**
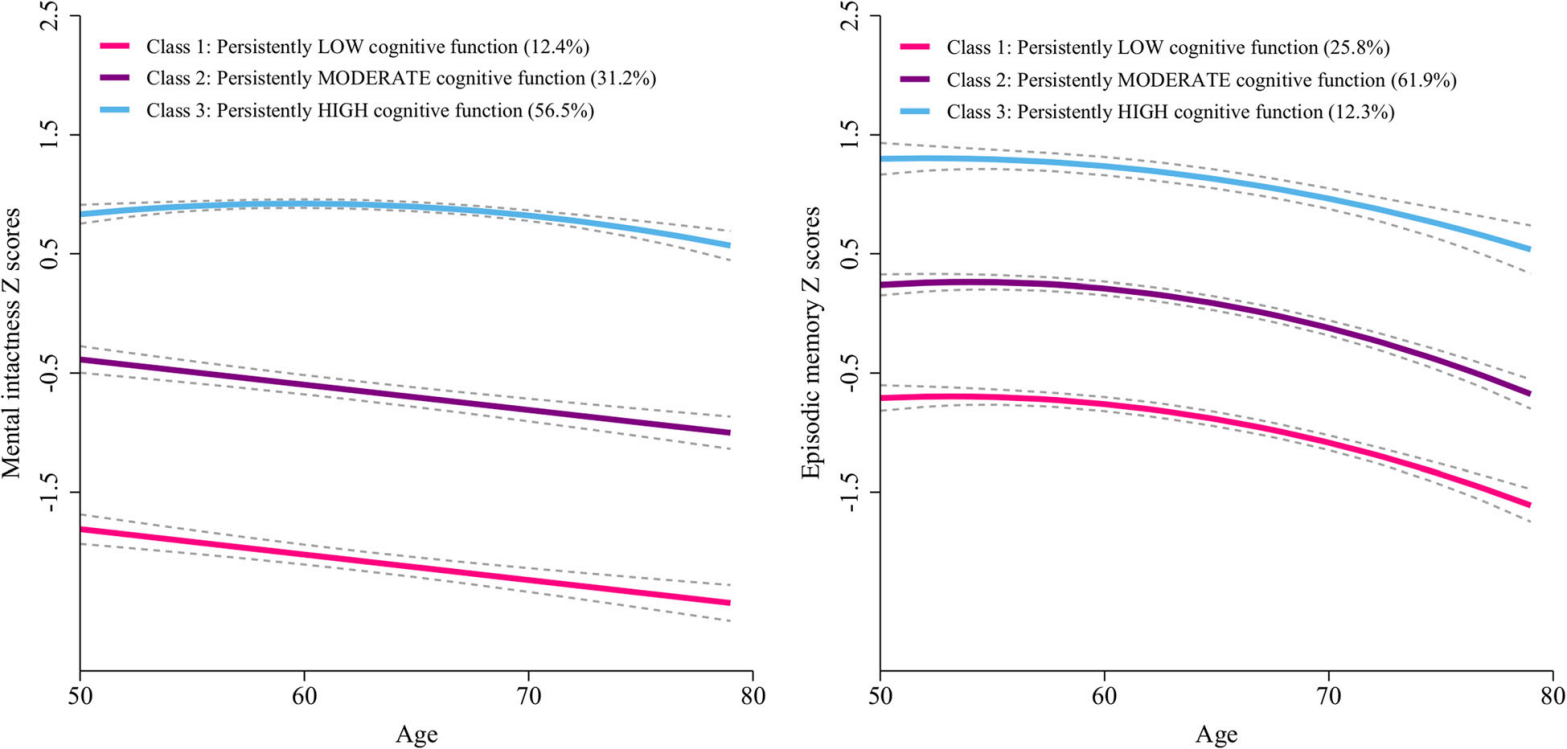
Mean trajectories of mental intactness scores (left) and episodic memory scores (right) by increasing age among older adults

We tested how many trajectories of cognitive function were optimal to explain the heterogeneity in the global cognitive scores in this population (Table 2). The BIC was lowest for the model with four trajectories (BIC = − 32,098.63); however, the average posterior probabilities were less than 0.7 for two trajectories groups. Thus, we identified the GBTM model with three trajectories as the optimal model.  Figure 2 shows three longitudinal patterns of cognitive function, plotted by current age at each visit, based on the global cognitive scores: class 1, “persistently low” (*n* = 1550, 18.9%); class 2, “persistently moderate” (*n* = 3194, 38.9%); and class 3, “persistently high” (*n* = 3460, 42.2%). The maximum likelihood estimates for the final three-group trajectory model are summarized in Table 3. The three group trajectories for domain of cognitive function were shown in Fig. 3.

**Table 2:** Fit statistics for global cognitive function group trajectories in middle-aged and older adults from CHARLS

Fit statistic	Number of classes					
	1	2	3	4	5	6
BIC*	− 35,903.71	− 32,658.89	− 32,120.03	− 32,098.63	− 32,099.94	− 32,116.00
AIC*	− 35,886.18	− 32,623.83	− 32,074.44	− 32,039.02	− 32,029.82	− 32,031.85
Class proportion^¶^	Class 1, 100%	Class 1, 38.29%	Class 1, 18.89%	Class 1, 12.49%	Class 1, 13.36%	Class 1, 13.24%
		Class 2, 61.71%	Class 2, 38.93%	Class 2, 21.54%	Class 2, 18.36%	Class 2, 17.61%
			Class 3, 42.17%	Class 3, 31.55%	Class 3, 20.40%	Class 3, 19.34%
				Class 4, 34.42%	Class 4, 36.26%	Class 4, 0.40%
					Class 5, 11.62%	Class 5, 36.18%
						Class 6, 13.23%
APP^‡^		Class 1, 0.92	Class 1, 0.87	Class 1, 0.80	Class 1, 0.79	Class 1, 0.79
		Class 2, 0.94	Class 2, 0.80	Class 2, 0.67	Class 2, 0.56	Class 2, 0.56
			Class 3, 0.89	Class 3, 0.67	Class 3, 0.50	Class 3, 0.48
				Class 4, 0.84	Class 4, 0.79	Class 4, 0.58
					Class 5, 0.48	Class 5, 0.67
						Class 6, 0.47

*CHARLS* China Health and Retirement Longitudinal Study, *AIC* Akaike’s information criterion, *BIC* Bayesian information criteria, *APP* average posterior probabilities

*A lower absolute value suggests a better model fit

^¶^No less than 5% of total count in a class

^‡^A higher value is better (preferably > 0.7 in a class)

**Table 3:** The final three-group trajectory model of global cognitive scores as function of age in middle-aged and older adults from CHARLS

Trajectory group	Parameter	Maximum likelihood estimates			
		Est.	SE	*z* value	*p* value
Class 1: persistently low cognitive function (*n* = 1550, 18.9%)	Intercept	− 5.04	1.21	− 4.157	< 0.001
	Linear (age)	1.38	0.38	3.592	< 0.001
	Quadratic (age^2^)	− 0.13	0.03	− 4.201	< 0.001
Class 2: persistently moderate cognitive function (*n* = 3194, 38.9%)	Intercept	− 6.02	0.98	− 6.153	< 0.001
	Linear (age)	2.05	0.31	6.615	< 0.001
	Quadratic (age^2^)	− 0.18	0.03	− 7.308	< 0.001
Class 3: persistently high cognitive function (*n* = 3460, 42.2%)	Intercept	11.35	6.13	1.853	0.064
	Linear (age)	− 5.26	2.93	− 1.795	0.073
	Quadratic (age^2^)	0.89	0.46	1.928	0.054
	Cubic (age^3^)	− 0.05	0.02	− 2.109	0.035

*CHARLS* China Health and Retirement Longitudinal Study, *Est.* parameter estimate, *SE* standard error of parameter estimate

### Trajectory sub-population characteristics

The baseline characteristics of the participants in each trajectory group for global cognitive function are presented in Table 4. Participants in the “persistently low” trajectory group were more likely to be older, be female, have lower levels of education and income, and have a high prevalence of depressive symptoms, restriction activities of daily diving, and visual or hearing impairments compared with those in the “persistently high” trajectory group.

**Table 4:** Baseline characteristics of the participants according to trajectories of global cognitive function in middle-aged and older adults from CHARLS

Characteristic	Trajectory of global cognitive function			*p* value
	Class 1, persistently low (*n* = 1550)	Class 2, persistently moderate (*n* = 3194)	Class 3, persistently high (*n* = 3460)	
Age (years), mean ± SD	60.45 ± 6.31	60.26 ± 6.41	59.77 ± 6.35	< 0.001
Male sex, *n* (%)	483 (31.2)	1677 (52.5)	2129 (61.5)	< 0.001
Educational level, *n* (%)				< 0.001
No formal education	1305 (84.2)	1590 (49.8)	656 (19.0)	
Primary school	175 (11.3)	910 (28.5)	926 (26.8)	
Middle or high school	68 (4.4)	660 (20.7)	1542 (44.6)	
College or above	2 (0.1)	34 (1.1)	336 (9.7)	
Married, *n* (%)	1316 (84.9)	2826 (88.5)	3168 (91.6)	< 0.001
Rural residence, *n* (%)	1175 (75.8)	2106 (65.9)	1625 (47.0)	< 0.001
High household income, *n* (%)	295 (19.0)	740 (23.2)	1316 (38.0)	< 0.001
Current smoker, *n* (%)	383 (24.7)	1134 (35.5)	1240 (35.8)	< 0.001
Current drinker, *n* (%)	389 (25.1)	1096 (34.3)	1371 (39.6)	< 0.001
Poor self-report of health, *n* (%)	552 (35.6)	906 (28.4)	649 (18.8)	< 0.001
Depressive symptoms, *n* (%)	775 (50.0)	1239 (38.8)	840 (24.3)	< 0.001
Restriction on ADL, *n* (%)	330 (21.3)	502 (15.7)	325 (9.4)	< 0.001
Visual impairment, *n* (%)	135 (8.7)	203 (6.4)	126 (3.6)	< 0.001
Hearing impairment, *n* (%)	159 (10.3)	254 (8.0)	187 (5.4)	< 0.001
Comorbidity, *n* (%)*				0.715
0	212 (13.7)	441 (13.8)	513 (14.8)	
1	362 (23.4)	765 (24.0)	814 (23.5)	
≥ 2	976 (63.0)	1988 (62.2)	2133 (61.6)	
BMI (kg/m^2^), mean ± SD	23.01 ± 3.71	23.41 ± 4.07	23.92 ± 3.85	< 0.001
Social activity scores, *n* (%)				< 0.001
0	991 (63.9)	2034 (63.7)	1894 (54.7)	
1–2	256 (16.5)	551 (17.3)	653 (18.9)	
≥ 3	303 (19.5)	609 (19.1)	913 (26.4)	
Intellectual activity scores, *n* (%)				< 0.001
0	1377 (88.8)	2605 (81.6)	2425 (70.1)	
1–2	123 (7.9)	438 (13.7)	632 (18.3)	
≥ 3	50 (3.2)	151 (4.7)	403 (11.6)	
Global cognitive scores, mean ± SD	7.50 ± 1.92	10.74 ± 2.36	14.49 ± 2.13	< 0.001
Mental intactness scores, mean ± SD	4.63 ± 1.85	7.28 ± 2.10	9.73 ± 1.57	< 0.001
Episodic memory scores, mean ± SD	2.86 ± 1.31	3.45 ± 1.39	4.76 ± 1.47	< 0.001

Abbreviations as in Table 1

### Baseline intellectual, social activity scores and cognitive trajectories

**Fig. 4:**
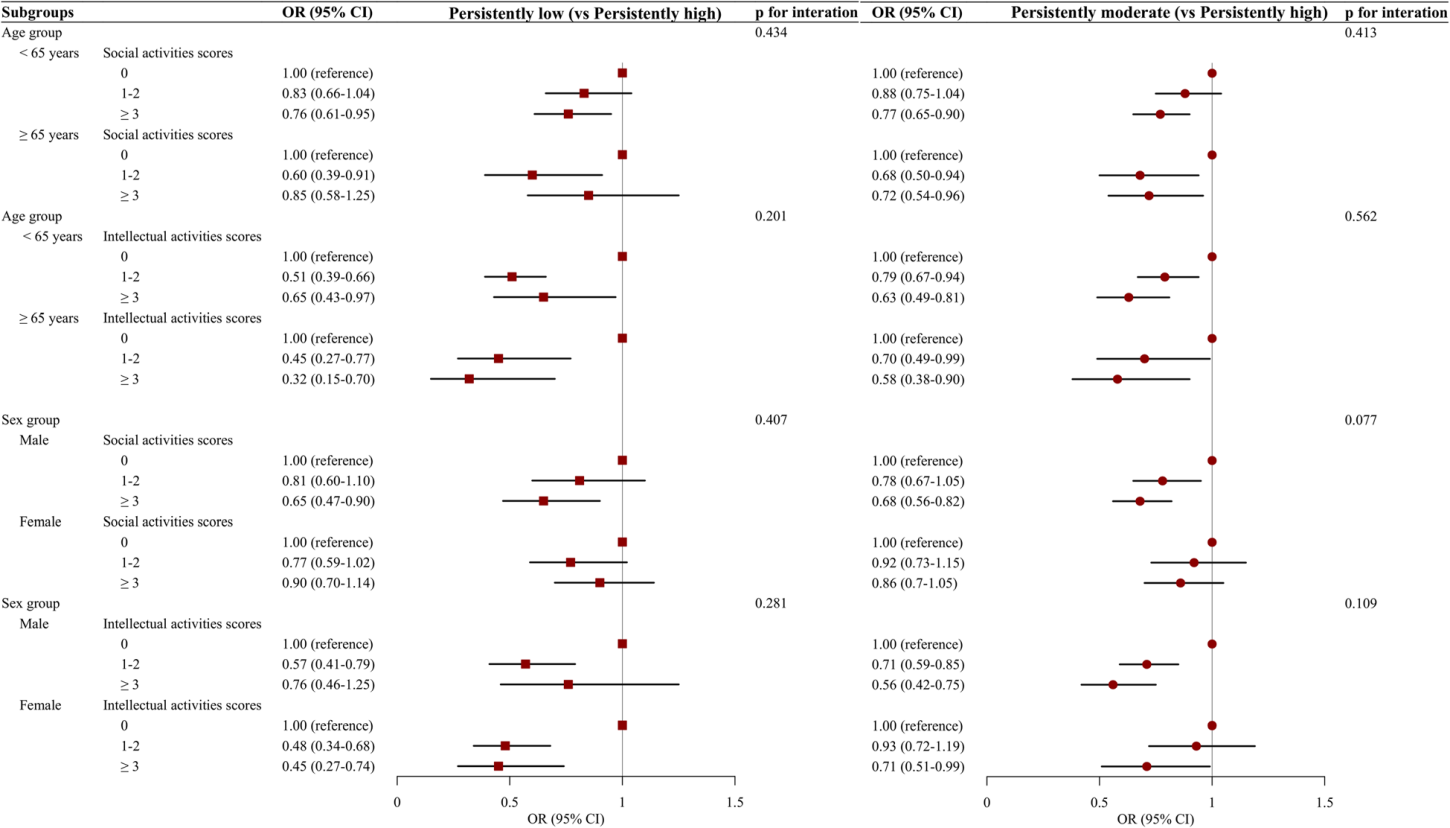
Stratified analysis by age group and sex for the association of associations of intellectual activities and social activities with the membership to cognitive function trajectory group

Table 5 summarizes the results from the multinomial regression examining intellectual, social activity scores associated with cognitive trajectory membership. Compared to participants who did not attend social activities (score = 0), adults who reported frequent participation in social activities (score ≥ 3) had better cognitive trajectories, with multivariable-adjusted OR (95% CI) for the “persistently low” and “persistently moderate” trajectories of global cognitive function of 0.79 (0.65–0.95) and 0.76 (0.66–0.87), respectively. The corresponding OR (95% CI) for frequent participation in intellectual activities (scores ≥ 3) were 0.54 (0.38–0.77) for “persistently low” cognitive function and 0.62 (0.50–0.77) for “persistently moderate” cognitive function. As shown in Fig. 4, the associations of social/intellectual activities and cognition trajectory group were similar between the younger (age < 65 years) and older (age ≥ 65 years) as well as male and female (all *p* values > 0.05 for interaction).

**Table 5:** Multinomial logistic regression analysis for the associations of intellectual activities and social activities with the membership to cognitive function trajectory group

	Persistently low (vs persistently high)		Persistently moderate (vs persistently high)	
	OR (95% CI)*	*p* value	OR (95% CI)*	*p* value
Global cognitive scores				
Social activity scores				
0	1.00 (reference)		1.00 (reference)	
1–2	0.77 (0.63–0.94)	0.011	0.84 (0.73–0.97)	0.020
≥ 3	0.79 (0.65–0.95)	0.011	0.76 (0.66–0.87)	< 0.001
Intellectual activity scores				
0	1.00 (reference)		1.00 (reference)	
1–2	0.49 (0.39–0.63)	< 0.001	0.78 (0.67–0.90)	0.001
≥ 3	0.54 (0.38–0.77)	0.001	0.62 (0.50–0.77)	< 0.001
Mental intactness scores				
Social activity scores				
0	1.00 (reference)		1.00 (reference)	
1–2	0.90 (0.72–1.12)	0.334	0.93 (0.80–1.07)	0.309
≥ 3	0.93 (0.76–1.15)	0.513	0.81 (0.71–0.93)	0.004
Intellectual activity scores				
0	1.00 (reference)		1.00 (reference)	
1–2	0.41 (0.31–0.55)	< 0.001	0.74 (0.63–0.86)	< 0.001
≥ 3	0.45 (0.28–0.71)	0.001	0.75 (0.60–0.95)	0.017
Episodic memory scores				
Social activity scores				
0	1.00 (reference)		1.00 (reference)	
1–2	0.58 (0.47–0.73)	< 0.001	0.67 (0.56–0.81)	< 0.001
≥ 3	0.60 (0.49–0.74)	< 0.001	0.72 (0.60–0.85)	< 0.001
Intellectual activity scores				
0	1.00 (reference)		1.00 (reference)	
1–2	0.76 (0.60–0.97)	0.026	0.98 (0.81–1.20)	0.863
≥ 3	0.42 (0.31–0.59)	< 0.001	0.67 (0.54–0.84)	< 0.001

*OR* odds ratio, *95% CI* 95% confidence intervals

*Adjusted for age at baseline (continuous), sex (male, female), education (no formal education, primary school, middle or high school, college or above), marital status (married, others), residence (urban, rural), household income (low, medium, high), smoking (yes, no), drinking (yes, no), body mass index (< 18.5, 18.5–23.9, 24.0–27.9, ≥ 28.0 kg/m^2^), self-report of health (good, fair, poor), comorbidity (0, 1, ≥ 2), depressive symptoms (yes, no), restriction on activities of daily living (yes, no), visual impairment (yes, no), and hearing impairment (yes, no)

### Non-response analyses

From the completed CHARLS cohort, 3375 individuals (27.4%) were excluded from this study because of incomplete baseline data or a confirmed diagnosis of dementia and/or Parkinson’s disease or cognitive impairment. Compared to those included in the current analyses, excluded participants were more likely to be women, living rural area, currently smoking and drinking, and having depressive symptoms and self-reported multimorbidity. Excluded participants also had lower education level and poorer cognitive function at baseline (Additional file 1: Table S1). An additional 759 individuals (6.2%), who were excluded because of loss to follow-up, also had higher levels of the major risk factors but had a good cognitive function at baseline (Additional file 1: Table S2).

### Sensitivity analyses

Firstly, given that trajectory analysis is more stable for participants with 3 or more observations over time, we conducted a sensitivity analyses by included participants with all three waves of cognitive function measures. The patterns of trajectory of global cognitive scores using completed all three waves’ cognitive function data (*n* = 6776) were similar to those from the main analyses: class 1, “persistently low” (*n* = 1272, 18.8%); class 2, “persistently moderate” (*n* = 2654, 39.2%); and class 3, “persistently high” (*n* = 2850, 42.1%) (Additional file 1: Fig. 1). The association between intellectual, social activity scores and global cognitive trajectory scores was similar results to those of the main analysis (Additional file 1: Table S3).

Secondly, mixed models for repeated measures were used to test the associations with frequency of social and intellectual activity as predictors (separate analyses) and global cognitive *Z* scores over time as an outcome. After adjusted for all potential confounders, we found that both more social activity score (*β* = 0.08, *z* = 6.28, *p* < 0.001) and intellectual activity score (*β* = 0.12, *z* = 7.38, *p* < 0.001) were associated with better cognitive function over study periods, and frequency of social activity × time interaction (*z* = − 3.06, *p* = 0.002) was statistically significant.

Lastly, cognitive change score at the end of the study periods was calculated by differences in global cognitive *Z* scores between wave 3 and wave 1. We used a beeswarm plot to demonstrate the differences in cognitive change score by difference level of social/intellectual activity and found that there was a slight difference in cognitive change score across each of the baseline social/intellectual activity score (Additional file 1: Fig. S2). Then, a generalized additive model was used to explore the association of cognitive score at baseline and cognitive change score by each of the social/intellectual activity score. We found that higher intellectual activity score contributed to delaying the onset of accelerated cognitive decline when taking the same baseline cognitive performance into consideration (Additional file 1: Fig. S3).

## Discussion

We identified three trajectory groups for global cognitive function among a nationally representative sample of 8204 middle-aged and older Chinese adults recruited from the CHARLS. We demonstrated that more frequent social and intellectual activities were both associated with a better cognitive performance trajectory over time.

The membership and shape of cognitive trajectories varied across different populations of older people [28–31]. In this population-based longitudinal study, three cognitive trajectories were identified. Our findings showed that there were clear differences in the baseline levels of global cognitive function but relatively small differences in the slope between the three trajectory groups. The “persistently high” trajectory is more desirable indicating that an individual has high cognitive score and maintains it at a high level throughout the lifetime. Overall, 42.2% of older Chinese people had persistently high cognitive function trajectories.

Consistent with previous studies, we found that older people with high levels of engagement in social or intellectual activities had more favorable subsequent cognitive function than those with low levels of engagement [13, 32–35]. A cross-sectional analysis based on the CHARLS data also showed that participation in social or intellectual activities was associated with better cognitive function [36]. The Cognitive and Lifestyle Activity Study for Seniors in Asia (CLASSA) showed that intellectual and physical activities, but not social activities, were cross-sectionally associated with better global cognition [14]. Similarly, in the Paquid cohort, engagement in social, physical, and intellectual activities was associated with a favorable cognitive trajectory over 20 years of follow-up [12, 37]. In the Health and Retirement Study, which used similar statistical methods, it was also shown that more social engagement in old age was associated with a lower risk of a declining cognitive trajectory [38]. As the first longitudinal study among Chinese older adults using trajectory analyses, our study contributed further knowledge of the association of social and intellectual activities with better cognitive trajectories over time in a Chinese population.

The strengths of this study are that it used well-validated measures of cognitive function and different types of leisure time activities. We followed a relatively large nationally representative cohort of middle-aged and older Chinese adults for a 4-year follow-up period with complete assessment of cognitive function. We also used advanced statistical models (namely, GBTM) to fit the cognitive aging trajectories. This approach helped identify groups of individuals who experienced similar levels and patterns of cognitive functions over time, while linear mixed models focus on mean population trajectories.

There are several limitations. First, although the frequencies and types of leisure activities were measured using a well-validated questionnaire, recall bias still existed. However, recall bias was very likely non-differential, which would bias the associations towards the null, which shows the robustness of our findings. Second, although the associations between social activities, intellectual activities, and cognitive aging trajectories were robust after adjustment for various demographic characteristics, socioeconomic status, health behaviors, and health conditions, residual confounding factors were not fully controlled, such as the APOE genotype [39]. Unfortunately, genotype data is not available in the CHARLS. However, the risk APOE e4 allele is less common in Asians compared to European populations [40]. According to a genomic study among 3679 Chinese, the frequency of e2 allele was 7.6%, e3 was 85.5%, and e4 allele was 6.9%. Therefore, the impact of the APOE status to our finding should be modest. Third, the no-response rate is high in the current analysis (27.4%). However, these excluded participants had lower education level and poorer cognitive function at baseline. If not missing, these participants would have been very likely in the consistently low cognition trajectory. Since both intellectual and social activities are less common among people with these characteristics, including these participants would have strengthen the identified associations, which indicates that our findings are robust. Fourth, intellectual and social activities were associated with better cognitive trajectory; however, reverse causality may exist. We have removed participants with very low cognitive scores at baseline, which would reduce the impact of reverse causality. In addition, social activities are less likely to be influenced by cognitive abilities. Fifth, participants in all trajectory groups had modest decline in cognitive functions. This may be due to short follow-up time. As the CHARLS continues to follow-up the participants, we would expect greater decline in cognitive function in these groups. Meanwhile, practice effects in the measurement of cognitive function may also be responsible. Since the same surveys were used in all follow-up visits, participants might get familiar with the cognitive tests and therefore performed better than expected in the follow-up surveys. Finally, our findings were based on an observational study and the population-based randomized control trials should be implemented to further explore the effect of social or intellectual activity on cognitive function in the future research.

## Conclusion

In conclusion, we found that participation in social activities and/or intellectual activities in midlife was independently associated with a favorable cognitive aging trajectory over time.

## Supplementary information

**Additional file 1: Table S1.** Comparison of baseline characteristics between participants included (*n* = 8204), and excluded due to incomplete baseline data or confirmed diagnosis of dementia and/or Parkinson’s disease or cognitive impairment (*n* = 3375). **Table S2.** Comparison of baseline characteristics between participants included (n = 8204) and excluded due to loss to follow-up (*n* = 759). **Table S3.** Multinomial logistic regression analysis for the associations of intellectual activities and social activities with the membership to global cognitive scores trajectory group. **Fig. S1.** Mean trajectories of global cognitive scores by increasing age among older adults among 6776 participants with completed all three waves cognitive function data. **Fig. S2**. The beeswarm plot demonstrating the differences in cognitive change score by difference level of social/intellectual activity. **Fig. S3.** The association of cognitive score at baseline and cognitive change score by each of the social/intellectual activity score.

## Data Availability

The CHARLS dataset is freely available to all bona fide researchers. Researchers can gain access to the data (http://charls.pku.edu.cn/en). Data can also be obtained on request (statguo@ccmu.edu.cn).
