# Supplementary material for: Associations between social and intellectual activities with cognitive trajectories in Chinese middle-aged and older adults: a nationally representative cohort study
Source: Alzheimers Res Ther. 2020 Sep 25;12:115. doi: 10.1186/s13195-020-00691-6 (PMC7519540; doi:10.1186/s13195-020-00691-6)
Supplement: Supplementary file 1 — Additional file 1: Table S1. Comparison of baseline characteristics between participants included (n = 8204), and excluded due to incomplete baseline data or confirmed diagnosis of dementia and/or Parkinson’s disease or cognitive impairment (n = 3375). Table S2. Comparison of baseline characteristics between participants included (n = 8204) and excluded due to loss to follow-up (n = 759). Table S3. Multinomial logistic regression analysis for the associations of intellectual activities and social activities with the membership to global cognitive scores trajectory group. Fig. S1. Mean trajectories of global cognitive scores by increasing age among older adults among 6776 participants with completed all three waves cognitive function data. Fig. S2. The beeswarm plot demonstrating the differences in cognitive change score by difference level of social/intellectual activity. Fig. S3. The association of cognitive score at baseline and cognitive change score by each of the social/intellectual activity score. [file 13195_2020_691_MOESM1_ESM.docx]

| Table S1. Comparison of baseline characteristics between participants included (n = 8204), and excluded due to incomplete baseline data or confirmed diagnosis of dementia and/or Parkinson’s disease or cognitive impairment (n = 3375) | | | |
| --- | --- | --- | --- |
| Characteristic | Included (n = 8204) | Excluded (n = 3375) | p for difference* |
| Age (years), mean ± *SD* | 60.09 ± 6.37 | 61.38 ± 7.00 | <0.001 |
| Male sex, *n* (%) | 4289 (52.3) | 1462 (43.3) | <0.001 |
| Educational level, *n* (%) |  |  | <0.001 |
| No formal education | 3551 (43.3) | 2151 (63.8) |  |
| Primary school | 2011 (24.5) | 572 (17.0) |  |
| Middle or high school | 2270 (27.7) | 562 (16.7) |  |
| College or above | 372 (4.5) | 87 (2.6) |  |
| Married, *n* (%) | 7310 (89.1) | 2878 (85.3) | <0.001 |
| Rural residence, n (%) | 4906 (59.8) | 2272 (67.3) | <0.001 |
| High household income, *n* (%) | 2450 (29.9) | 753 (22.3) |  |
| Current smoker, *n* (%) | 2757 (33.6) | 653 (22.3) | <0.001 |
| Current drinker, *n* (%) | 2856 (34.8) | 1000 (29.8) | <0.001 |
| Poor self-report of health, *n* (%) | 2107 (25.7) | 1289 (38.3) |  |
| Depressive symptoms, *n* (%) | 2854 (34.8) | 2194 (65.0) | <0.001 |
| Restriction on ADL, *n* (%) | 1157 (14.1) | 813 (24.1) | <0.001 |
| Visual impairment, *n* (%) | 464 (5.7) | 277 (8.2) | <0.001 |
| Hearing impairment, *n* (%) | 600 (7.3) | 412 (12.2) | <0.001 |
| Comorbidity ≥ 2, *n* (%) | 5097 (62.1) | 2446 (72.5) | <0.001 |
| BMI (kg/m^2^), mean ± SD | 23.54 ± 3.92 | 22.76 ± 3.89 | <0.001 |
| Global cognitive scores, mean ± *SD* | 11.71 ± 3.43 | 3.18 ± 0.93 | <0.001 |
| Mental intactness scores, mean ± *SD* | 7.82 ± 2.64 | 1.77 ± 0.96 | <0.001 |
| Episodic memory scores, mean ± *SD* | 3.89 ± 1.61 | 1.41 ± 0.90 | <0.001 |
| *The differences between participants included and excluded were tested using the t-test or chi-square test. | | | |

| Table S2. Comparison of baseline characteristics between participants included (n = 8204) and excluded due to loss to follow-up (n = 759) | | | |
| --- | --- | --- | --- |
| Characteristic | Included (n = 8204) | Loss to follow-up (n = 759) | p for difference* |
| Age (years), mean ± *SD* | 60.09 ± 6.37 | 60.43 ± 7.14 | 0.16 |
| Male sex, *n* (%) | 4289 (52.3) | 418 (55.1) | 0.14 |
| Educational level, *n* (%) |  |  | <0.001 |
| No formal education | 3551 (43.3) | 231 (30.5) |  |
| Primary school | 2011 (24.5) | 172 (22.7) |  |
| Middle or high school | 2270 (27.7) | 271 (35.8) |  |
| College or above | 372 (4.5) | 84 (11.1) |  |
| Married, *n* (%) | 7310 (89.1) | 670 (88.3) | 0.48 |
| Rural residence, n (%) | 4906 (59.8) | 246 (32.4) | <0.001 |
| High household income, *n* (%) | 2450 (29.9) | 265 (34.9) | <0.001 |
| Current smoker, *n* (%) | 2757 (33.6) | 249 (32.8) | 0.67 |
| Current drinker, *n* (%) | 2856 (34.8) | 253 (33.3) | 0.41 |
| Poor self-report of health, *n* (%) | 2107 (25.7) | 219 (28.9) | 0.16 |
| Depressive symptoms, *n* (%) | 2854 (34.8) | 251 (33.1) | 0.34 |
| Restriction on ADL, *n* (%) | 1157 (14.1) | 144 (19.0) | <0.001 |
| Visual impairment, *n* (%) | 464 (5.7) | 45 (5.9) | 0.76 |
| Hearing impairment, *n* (%) | 600 (7.3) | 44 (5.8) | 0.12 |
| Comorbidity ≥ 2, *n* (%) | 5097 (62.1) | 613 (80.8) | <0.001 |
| BMI (kg/m^2^), mean ± SD | 23.54 ± 3.92 | 23.77 ± 4.37 | 0.20 |
| Global cognitive scores, mean ± *SD* | 11.71 ± 3.43 | 12.25 ± 3.50 | <0.001 |
| Mental intactness scores, mean ± *SD* | 7.82 ± 2.64 | 8.24 ± 2.59 | <0.001 |
| Episodic memory scores, mean ± *SD* | 3.89 ± 1.61 | 4.01 ± 1.73 | 0.062 |
| *The differences between participants included and excluded were tested using the t-test or chi-square test. | | | |

| Table S3. Multinomial logistic regression analysis for the associations of intellectual activities and social activities with the membership to global cognitive scores trajectory group | | | | | |
| --- | --- | --- | --- | --- | --- |
|  | Persistently low  (vs Persistently high) | |  | Persistently moderate  (vs Persistently high) | |
|  | OR (95% CI)* | *p* value |  | OR (95% CI)* | *p* value |
| Social activities scores |  |  |  |  |  |
| 0 | 1.00 (reference) |  |  | 1.00 (reference) |  |
| 1-2 | 0.82 (0.66-1.03) | 0.083 |  | 0.88 (0.75-1.03) | 0.104 |
| ≥ 3 | 0.87 (0.71-1.07) | 0.182 |  | 0.79 (0.68-0.92) | 0.002 |
| Intellectual activities scores |  |  |  |  |  |
| 0 | 1.00 (reference) |  |  | 1.00 (reference) |  |
| 1-2 | 0.47 (0.36-0.61) | <0.001 |  | 0.74 (0.63-0.87) | <0.001 |
| ≥ 3 | 0.45 (0.30-0.68) | <0.001 |  | 0.58 (0.46-0.75) | <0.001 |

Note: OR = Odd Ratio, 95% CI = 95% confidence intervals.

*Adjusted for age at baseline (continuous), sex (male, female), education (no formal education, primary school, middle or high school, college or above), marital status (married, others), residence (urban, rural), household income (low, medium, high), smoking (yes, no), drinking (yes, no), body mass index (< 18.5, 18.5-23.9, 24.0-27.9, ≥ 28.0 kg/m2), self-report of health (good, fair, poor), comorbidity (0, 1, ≥ 2), depressive symptoms (yes, no), restriction on activities of daily living (yes, no), visual impairment (yes, no), and hearing impairment (yes, no).

Fig. S1 Mean trajectories of global cognitive scores by increasing age among older adults among 6776 participants with completed all three waves cognitive function data.


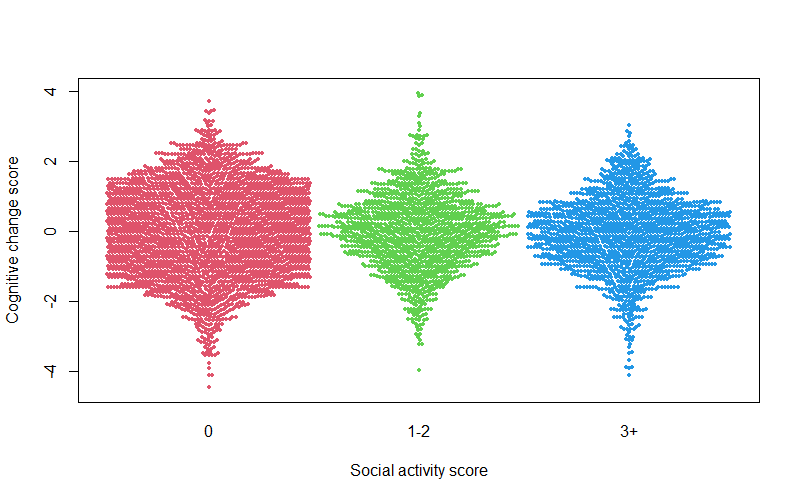


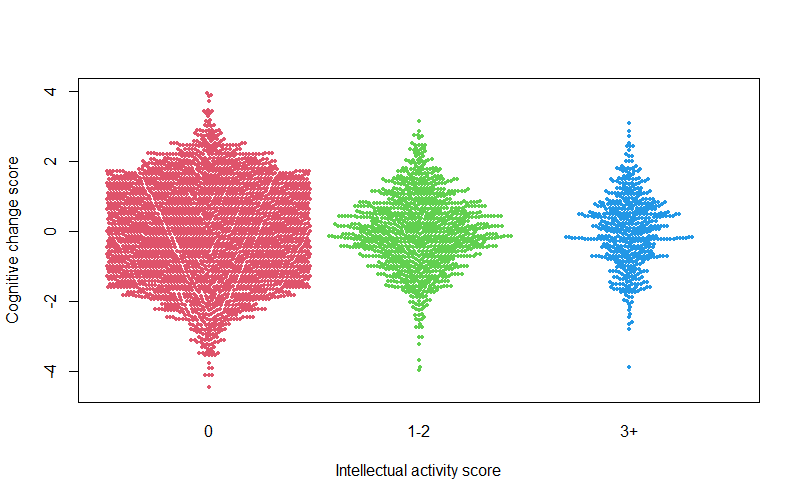


Fig. S2 The beeswarm plot demonstrating the differences in cognitive change score by difference level of social/intellectual activity


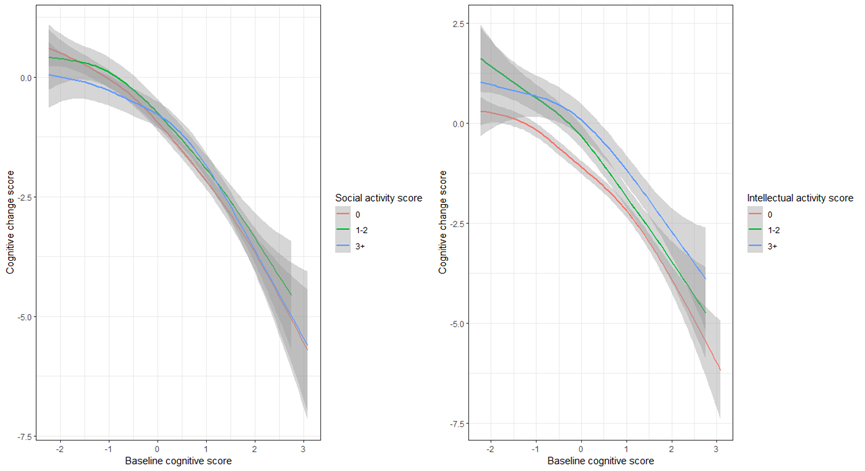
Fig. S3 The association of cognitive score at baseline and cognitive change score by each of the social/intellectual activity score
